# Ten simple rules for teaching yourself R

**DOI:** 10.1371/journal.pcbi.1010372

**Published:** 2022-09-01

**Authors:** Jake Lawlor, Francis Banville, Norma-Rocio Forero-Muñoz, Katherine Hébert, Juan Andrés Martínez-Lanfranco, Pierre Rogy, A. Andrew M. MacDonald

**Affiliations:** 1 McGill University, Department of Biology, Montréal, Québec, Canada; 2 Québec Centre for Biodiversity Science, Montréal, Québec, Canada; 3 Université de Montréal, Département de sciences biologiques, Montréal, Québec, Canada; 4 Université de Sherbrooke, Département de biologie, Sherbrooke, Québec, Canada; 5 University of Alberta, Department of Biological Sciences, Edmonton, Alberta, Canada; 6 University of British Columbia, Department of Zoology and Biodiversity Research Centre, Vancouver, British Columbia, Canada; Dassault Systemes BIOVIA, UNITED STATES

## Introduction

Statistical and scientific programming has quickly become a necessary skill in the sciences, often conducted in the programming language, R [1,2]. However, formal education in programming is not always prioritized in natural science degrees and may not be an expected prerequisite for students interested in careers or graduate degrees in fields like ecological or biological sciences. In fact, in a recent survey of science educators, the vast majority of respondents rated data skills (analysis and visualization) as *extremely* or *very important* for undergraduate students, but respondents particularly from bachelors-granting institutions also listed “outside of coursework” as the *most likely* place for students to learn such skills [3].

While this trend appears to be changing in recent years [4,5,6], many students and early-career scientists are still left with the task of learning quantitative and programming skills largely in a self-directed manner in order to meet their academic or professional goals. This “learning to program” hurdle is so common in biological sciences that it has even been the focus of other *10 Simple Rules* articles [7,8]. Here, we focus specifically on self-learning the programming language, R [9], and attempt to provide a roadmap for new R users unable to see a clear path ahead.

Importantly, we write this list of strategies and resources not as the voice of authority of the vast and diverse universe of R users, but simply as a group of quantitative-minded mostly ecologists, generally located in universities across Canada, who have all learned R largely without formal instruction. Here, we share the tips and techniques that proved helpful along our own R self-teaching journeys in the hopes that we can make the process less intimidating for others. These 10 rules will not provide technical instruction for using R—as there are many wonderful and thorough resources for that (for instance, [10])—but rather provide a nonexhaustive list of practical strategies for building or honing R programming skills.

## Rule 1: Prepare for a steep learning curve

**Fig 1:**
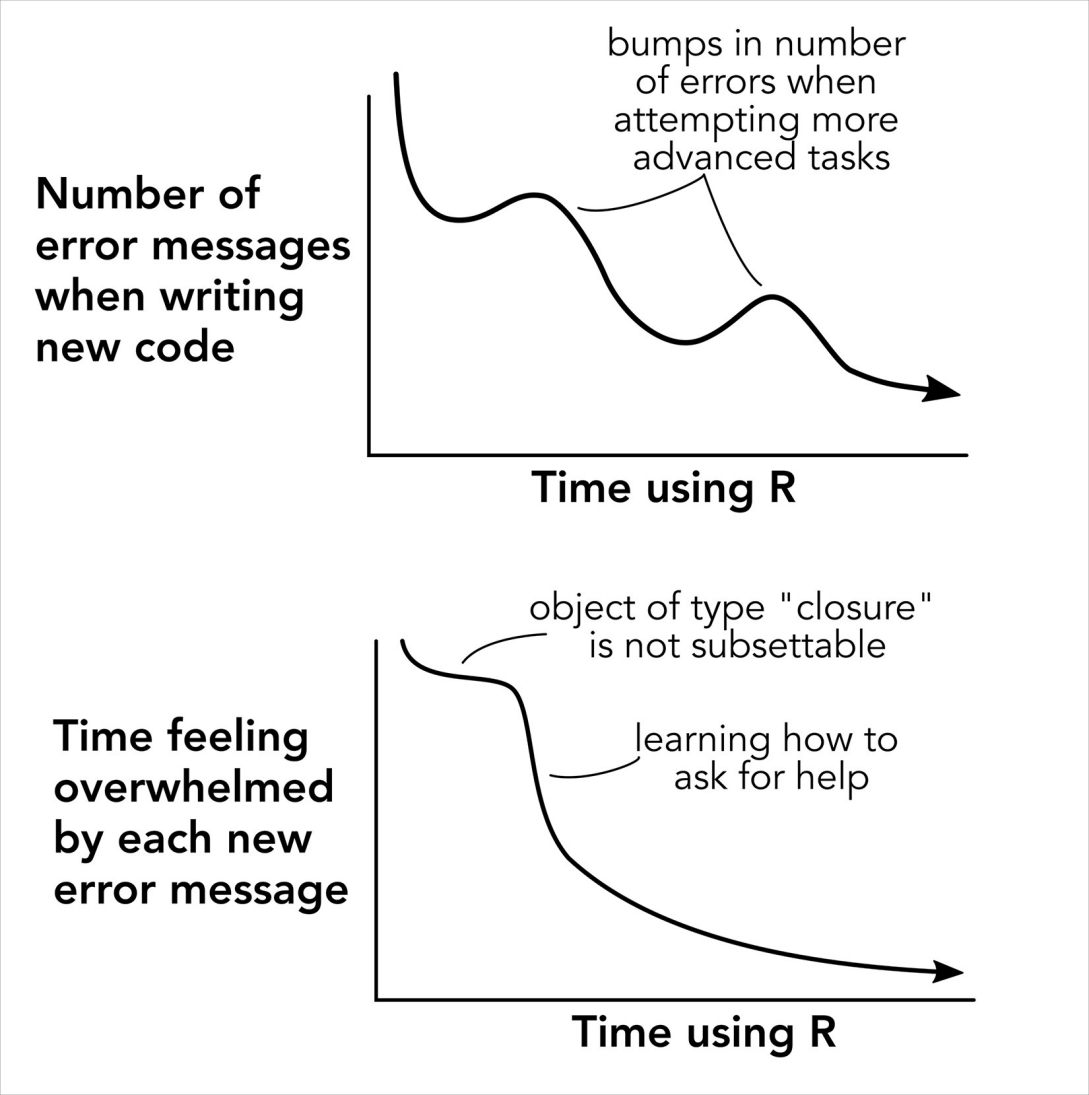
Proficiency in R isn’t about never making mistakes, but becoming comfortable encountering and solving them when you do.

Learning R is learning a new language; you are entering a new world of vocabulary, grammar, syntax, and maybe even a new style of thinking. That’s a lot of things to get used to! Learning R is difficult because it involves making mistakes often, working to identify those mistakes, and eventually learning how to fix them. Remember that you will never be perfect—even the most experienced R users still make mistakes, forget function arguments, or turn to internet searches to refresh on tasks they may have completed many times. Fluency in R is not about never receiving error messages, but feeling capable of fixing them when you do (Fig 1). Of course, some bugs occur not due to mistyped code, but instead due to misunderstandings of function arguments or outputs. While avoiding error messages is a great first step, carefully reading documentation and running code one line at a time are the best ways to ensure your scripts are completing their intended tasks.

## Rule 2: Take the time to read a book

A great way to learn proper programming practices is by reading books. An advantage of books is that they often represent an expert voice, the skill of the community, or both. Most good books for learning programming in R will contain code examples that you can use to sharpen your skills. Whichever book you choose, run the code examples and examine the outputs. When books don’t have answers in the back, it is helpful (and more fun!) to start or join a reading group (see Rule #8: Join the R community). Attempt the exercises with your colleagues, and use each other as a network to ask questions, compare approaches, and group-think through complicated tasks. Textbook solutions are also regularly shared online on personal blogs or websites. Posting your own solutions online will allow you to get feedback and will benefit the broader R community (see Rule #9).

R users are lucky to have an abundance of high-quality published books as learning resources, including over a dozen that are featured on the RStudio website (https://www.rstudio.com/resources/books/), and countless more authored by knowledgeable R contributors. Some of our favorite books for studying R include “R for Data Science” [10] and “Advanced R” [11], which present the modern approach to R via the *tidyverse* (see Rule #5 about style). Depending on your reason for learning R, it may also be helpful to follow books that cover more specific topics that align with your interests. For example, if you plan to use R for advanced statistics, you may turn to books like “Mixed-effects models and extensions in ecology with R” [12], or “Generalized Additive Models: An Introduction with R” [13], which each encourage a powerful understanding of core programming and statistical skills. If you plan to use R for communicating results through high-quality web graphics, you may be interested in “Interactive web-based data visualization with R, plotly, and shiny” [14], which covers introductory-to-advanced web-based data visualization techniques. Specialty books exist in various disciplines and can be wonderful resources for advancing field-specific skills.

## Rule 3: Use free resources

The monetary cost of learning a new programming language can be intimidating. How many books should you purchase? Which coding courses should you take? Fortunately, many high-quality R resources are available online for free and cover everything you need to know to get yourself started. For instance, many ebooks are available for free online, including those featured on the RStudio site (see Rule #2). For quicker referencing, RStudio also provides several single-page *cheatsheets*, each covering the basics of one specific package or programming task that can serve as great reminders.

There are also many free courses on R, statistics, and data science on sites like Coursera, edX, and freeCodeCamp, free training materials from The Carpentries, and even free video tutorials on sites like YouTube and Twitch. Some regional and global R groups such as R-Ladies and ROpenSci (see Rule #8 for more) offer blog posts, workshops, and resources in multiple languages. A list of R-Ladies blogs is maintained here (https://github.com/rladies/awesome-rladies-blogs); some of our favorites include those of Julia Silge and Danielle Navarro. Other curated lists of free resources can be found on the inSileco blog and the r-directory website. Whether it’s through blogs, interactive tutorials, or video lessons, if there is one online format that works best for you, there are likely R resources in that format—you may just have to search a bit to find them.

For more field-specific training in R techniques, many institutions offer free tutorials, workshops, and classes online. For example, the Quebec Centre for Biodiversity Science (QCBS) R Workshop Series offers introductory and advanced workshops on data visualization, linear models, multivariate analyses, and more, in both English and French, with freely available slides, code, and companion books on their website. The Coding Club from the University of Edinburgh offers a wide breadth of courses for ecologists and environmental scientists, ranging from data manipulation and statistics to geospatial analysis and machine learning. EcoDataScience, a group centered at the University of California Santa Barbara, offers skillshares and training on a range of ecology-relevant R techniques. These are only a few examples of quality learning groups; make sure to check with your institution, company, or colleagues for recommendations that may be most relevant or most convenient for you.

## Rule 4: Build skills with low-pressure projects

If you have the time and flexibility to do so, one great way to build skills is to practice R through projects that are “just for fun.” Want to learn to map with shapefiles? Try making a poster of your favorite city to print as wall art [15]. Data scraping through APIs? Try identifying common qualities among your most-played artists on Spotify [16]. Text analysis? Try to use R’s text mining packages to compare sentiment associations of your favorite books or TV shows [17,18]. Customized data visualization? Try using ggplot shapes and aesthetics to replicate your favorite piece of art (see #RecreationThursday on Twitter) [19].

Just-for-fun projects can be incredibly valuable environments for building crucial skills. The low-stakes environment will take the pressure off of you to succeed, but when you do, will give you new products to share, and working code to which you can refer next time you are attempting a similar task. Participation in just-for-fun community events, Twitter challenges, or R coding contests (see Rules #8 and #9 for more) will help you build solid foundations that will open doors in your programming path, all while doing projects that you enjoy!

If you prefer to keep your R learning to more “on-task” efforts, you can make low-pressure additions to your existing projects in order to gain new skills. Try practicing HTML rendering by adding custom text annotations to your existing figures [20] or upgrading your usual workflow by creating a Shiny app for interactive data exploration. Finding opportunities to broaden your skillset while improving your existing work will help you to become a more well-rounded R user.

## Rule 5: Adopt good practices and be consistent

Cultivating and maintaining an approach to coding and a system of organization for your projects will help your code to be clear and consistent for you and others.

When beginning coding projects, consider your mindset. For example, if you are most comfortable beginning new tasks with all future steps carefully planned, you may benefit from starting your projects with *pseudocode*. Pseudocode is a list of plain language descriptions for operations you plan to complete, which you will write out in a document and later translate line by line into code. A benefit of this method is that it allows you to conceptualize your project from start to finish and provides a discrete goal for each line of code that you will write. If you are more comfortable learning by doing, you may prefer to write code directly, observing the output of each line and commenting in the text what your last line completed, reaching the end goal in smaller increments. In both cases, it is important to comment your code throughout to ensure that future-you can understand what present-you was thinking.

Next, you will need to decide what *style* to use while coding. A coding style, in this context, is a collection of specific functions, packages, and syntax strategies that you use when writing your code. If you think of R as a language, you might think of your style as a dialect; two styles may look different, but their meaning is the same. In R, the main style divide usually revolves around whether you will use style typical of *Base R*, or adopt R’s *piped* approach, and other *tidyverse* principles (Table 1). Though these styles are not mutually exclusive, they imply different sets of functions and usually suit different syntax strategies to structure your code. Depending on your background, you may prefer one syntax over the other. For example, if you have experience coding in programs like C++ or Java, a sequential or nested syntax may look familiar to you, whereas piped functions might make your code read more similarly to a sentence written in English (see *tidyverse* style guide: https://style.tidyverse.org/).

**Table 1:** Examples of 3 styles of code, each changing an *object* (such as a data frame or a vector), using 2 *verbs* (functions that manipulate that object), and 2 *arguments* specifying those verbs (such as applying that function to only section X of the object). If these look unintelligible to you, that’s okay! They are only meant to show that in R, there are different syntax strategies to complete the same tasks. If one looks more interpretable than the others, then great—you have found your coding style to begin!

Sequential Syntax	Nested Syntax	Piped Syntax
object_step1 <- verb1(object, argument1) object_end <- verb2(object_step1, argument2)	object_end <- verb2( verb1(object, argument1 ), argument2 )	object_end <- object |> verb1(argument1) |> verb2(argument2)

In most cases, any style is fine as long as it works for you. Of course, matching the code style that is most common among your collaborators or standard in your discipline can be helpful for reading, sharing, and troubleshooting code. There may also be times when you need to switch styles to best complete a certain task, but choosing to be stylistically consistent whenever possible will help ensure that your code is interpretable by you and others.

Finally, it’s important to place your files into a rigorous directory structure. The R ecosystem offers many tools to facilitate project organization. Most integrated development environments (IDEs) have a concept of a “project directory”; this is true of the leading R IDE, RStudio, and also of most others (for instance, VScode and Atom). You can also use packages, such as **here** [21], to simplify the file pathway naming schemes within your project directory. Depending on your field, your folder organization structure may look a bit different, but critically thinking through your system will simplify your life as your project list grows. In addition, maintaining proper project organization will help you in learning the practice of “version control,” a system of tracking changes and backing up code that is becoming a professional standard across scientific fields. For more on file and code organization, see [22].

## Rule 6: Use CRAN’s Task View

Most of R’s functions are bundled together in task-specific *packages*, but it can be difficult to know which packages exist and to understand which are most applicable to certain tasks. In R, the main repository for user-developed packages is CRAN, the Comprehensive R Archive Network. While packages can come from other sources as well, CRAN packages have the perks of ease of access through R’s **install.packages()** function and assurance that the package has been tested on multiple platforms.

When searching for new packages needed to complete specific tasks, a great place to start is CRAN’s Task View (https://cran.r-project.org/web/views/). Task View allows users to browse published packages by topic, including multivariate statistics, spatiotemporal analyses, meta-analyses, and more. The Task View browser lists relevant packages for each topic and provides links to extended documentation of each featured package. This resource is useful for R beginners, as well as more experienced R users who are searching for ways to tackle new challenges.

## Rule 7: Ask for help (and help others)

When self-learning R, you may encounter problems that you do not yet know how to solve on your own. In these cases, asking for help may save you a considerable amount of time or a moderate amount of headache. Knowing how to ask for help in targeted ways will allow you to pinpoint the source of the problem, and, ideally, help you to avoid similar problems in the future.

When you first encounter a problem, copying and pasting errors in your search engine will likely lead you to websites like Stack Overflow, GitHub, or R-bloggers. More often than not, someone will have had the same issue as you and will have likely reached a solution that you can use. Otherwise, you may need to post your own call for help, in which case there are certain guidelines that will make your post more efficient.

When asking for help, reproducible examples are essential. You should aim to demonstrate your issue as minimally as possible, and will need to spend some time isolating the problematic code before posting your question. It’s most helpful to generate a toy dataset in your example or to use a dataset that is built into R (see **data()** in R) and incrementally strip away the parts of your code that are not relevant to the issue you want to solve. You should also accompany your minimal reproducible example with information about your R session and relevant package versions. All of these steps can be facilitated with the **reprex** package [23].

As your skills improve, you might find yourself in a position where you can help others. Remember that answering questions on places like Twitter or Stack Overflow is a great way to give back to the R community. You can even go back and answer questions that you yourself asked in the past, which will mark your post as “resolved,” and will leave a working solution for the next person who encounters the same problem.

## Rule 8: Join the R community

One of the best ways to build fluency in R is to learn it with others. The R community is vibrant and prolific, with conferences, meetups, and regular online events for learning R skills. One great place to start is to search in your area for local UseR or RLadies groups, each with chapters around the world, which usually host meetings for skillshares, lectures, and coding practice sessions.

If you are not near an R group location, you can also join the R community virtually. The R for Data Science (R4DS) community (based on Hadley Wickham and Garrett Grolemund’s book of the same name) offers space for learners and mentors to share R skills and work collaboratively. The R4DS Slack channel has over 10,000 members, with discussion channels where members can ask for help, share wins, network, and more.

**Fig 2:**
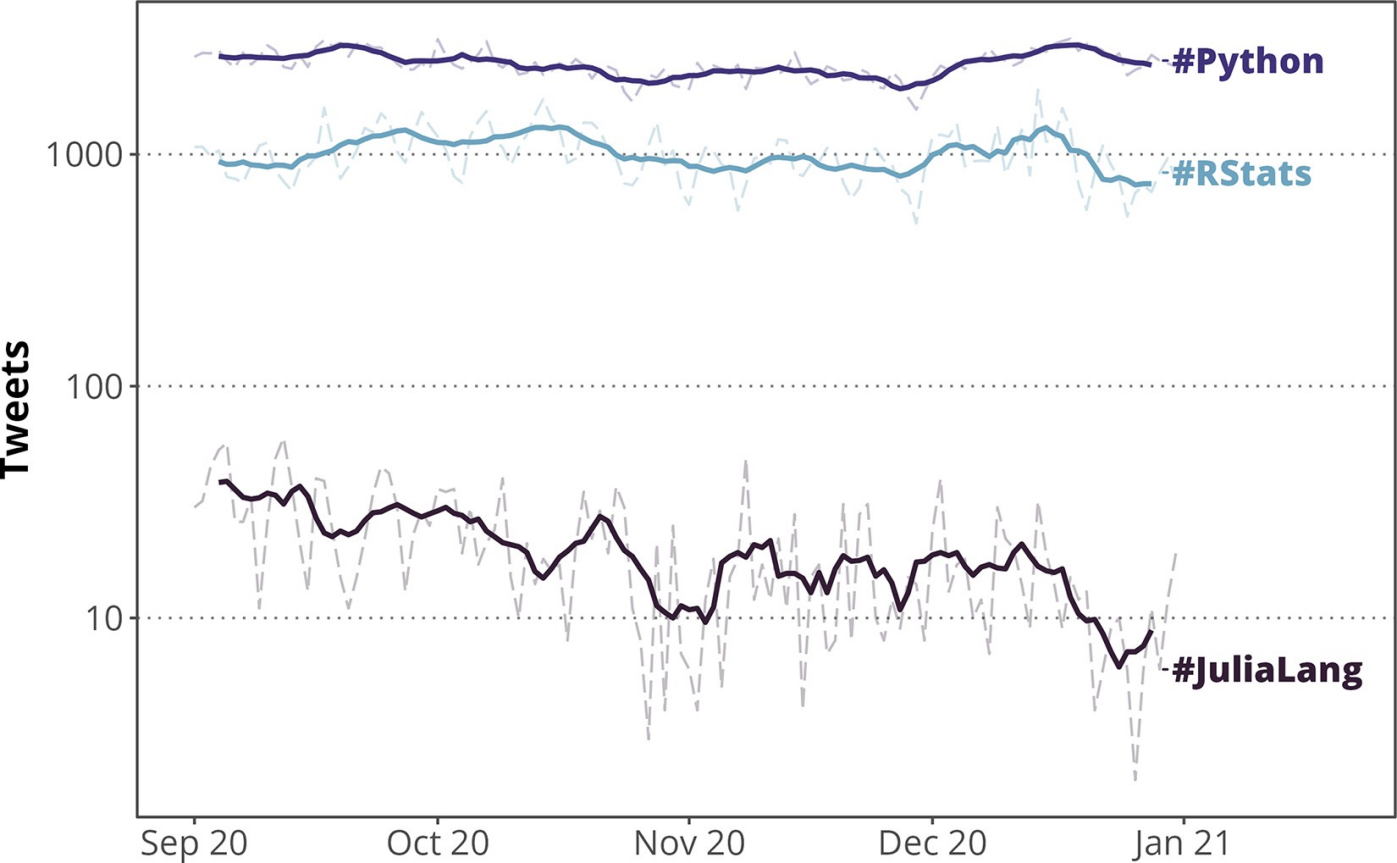
Popularity of Twitter discussion for some common scientific programming languages. While not as popular as #Python, #RStats appeared in around 1,000 tweets per day on average at the end of 2020. Solid lines show 7-day rolling averages; transparent dashed lines show daily tweet counts. Tweets were collected from September 1, 2020 to December 31, 2020, using the R package, **academictwitteR** [24]. We found a total of 124,852 Tweets with the #rstats hashtag, 303,388 with the #python hashtag, and 2,522 with the #julialang hashtag, within this 4-month period.

If you are not part of any specific group, the R community is also active on many social networks. R is commonly discussed on Twitter with the #RStats hashtag (Fig 2); a great entry point to the community could be following accounts like @rstudio, @Rbloggers, @icymi_r, @RLadiesGlobal, @R4DS, and @rOpenSci. Both general and field-specific R groups also exist on sites like Facebook (for instance, Ecology in R) and Reddit. You can even join online events like the data science show “SLICED” on Twitch, to watch experts compete in R programming challenges (among other languages). Following prolific R bloggers can also be a great way to stay up-to-date with R news; a few of our favorite R bloggers include Maëlle Salmon, Jacqueline Nolis, and Miles McBain. Wherever you end up, finding a community online can be a great way to learn about new features, stay informed about recent packages, come across tips and tricks that you may not see anywhere else, and most importantly, stay motivated to keep coding!

## Rule 9: Read others’ code, and share yours

R’s open-source culture comes with plentiful resources for code sharing. Reading and running code from publicly available sources can be incredibly valuable in discovering new functions, optimizing processing speeds, and learning from experts. Some R projects, such as the #TidyTuesday R social data visualization project, encourage code sharing online to help users to gain and hone skills [25,26]. Others, such as the annual RStudio Shiny Contest, provide an outlet for friendly competition of user-made R products with code freely available at the end, allowing users to read, download, and replicate award-winning applications. If you find a particularly inspiring R product with code provided, run the code yourself line by line to understand the exact contribution of each line or function. You might learn a new function that you can apply to your projects or that there are many ways of completing the same task.

Published papers provide another source of R code for more research-centric purposes. Many academic journals now require publicly available data and code to be included with publications, many of which, especially in the natural sciences, are written in R. By downloading materials from published literature, you can learn how experts in your field conduct their analyses, which packages they use, and how they organize their code.

Of course, code sharing is a two-way street; in addition to accessing publicly available materials from others, you should also share your own. Common platforms for code sharing include GitHub, GitLab, and Open Science Framework (OSF) (for Git and GitHub, see [27]). Sharing your R code online will further the open nature of the R community and help you be a more attentive code writer. Posting your code can be intimidating, but remember that available code will always be more valuable to the scientific community than unavailable code [28]. Don’t stress too much if your code isn’t completely optimized or totally clean. If it runs and it might be able to teach someone something new, it’s worth sharing.

## Rule 10: Don’t box yourself in

R is a wonderful tool that can be instrumental for statistics, data manipulation, visualization, and more, but it doesn’t have to be the end of your programming journey. Fluency in R will help you gain skills that apply to other programs, languages, or fields in the future. For example, designing User Interfaces (UIs) for Shiny Applications will help you build foundations of front-end web development, R’s various text plugins (such as the **ggtext** package [20]) will help you practice HTML syntax, and R’s vector operations (**apply()** and **purrr::map()** function families) will build conceptual frameworks to transfer to other programming languages such as Julia or Python. This works both ways: If you have developed skills in other programming languages, they will help you in your journey toward learning R.

As you continue applying your programming skills to a wider range of tasks, you may find that for some tasks, different tools would be more efficient or appropriate. In these cases, the confidence and skills you built while learning R may be a useful springboard for your next coding endeavor. The knowledge you will gain by learning R—and the experience you will have from self-teaching it—will benefit you far past your task at hand.

## Conclusions

Teaching yourself new skills can be hard. It can be a process rife with frustration, self-doubt, and low motivation to continue. The 10 rules we list here are our best strategies to overcome these challenges, master new techniques, and maybe even have some fun along the way! We hope that these rules will help new R users, be they graduate students, hobbyists, or established researchers eager to learn a new tool. We do not insist that readers try *all* of these rules at once, nor that they limit themselves to these ten alone. Learning R is a very individual process—you just have to find the methods that work best for you.
